# Biotechnological Production and Characterization of Extracellular Melanin by *Streptomyces nashvillensis*

**DOI:** 10.3390/microorganisms12020297

**Published:** 2024-01-30

**Authors:** Odile Francesca Restaino, Paola Manini, Talayeh Kordjazi, Maria Laura Alfieri, Massimo Rippa, Loredana Mariniello, Raffaele Porta

**Affiliations:** 1 Department of Chemical Sciences, University of Naples Federico II, Via Cintia 4, 80126 Naples, Italy; odilefrancesca.restaino@unina.it (O.F.R.); paola.manini@unina.it (P.M.); talayeh.kordjazi@unina.it (T.K.); marialaura.alfieri@unina.it (M.L.A.); raffaele.porta@unina.it (R.P.); 2 Institute of Applied Sciences and Intelligent Systems “E. Caianiello” of CNR, Via Campi Flegrei 34, 80078 Pozzuoli, Italy; massimo.rippa@isasi.cnr.it

**Keywords:** antioxidant, melanin, metal-chelation, UV-light protection, *Streptomyces nashvillensis* DSM 40314

## Abstract

Melanins are pigments employed in food, cosmetic, and textile industries, manufactured by extraction from cuttlefishes. Their biotechnological production by Streptomycetes, instead, has been poorly investigated so far. In this paper, for the first time, the strain *Streptomyces nashvillensis* DSM 40314 was tested as an extracellular melanin producer by investigating the influence of diverse temperatures (26, 28, and 30 °C) and pH values (6.0 and 7.0) on bacterial growth, melanin production, and on the activity of the secreted tyrosinase, the first enzyme of the pigment biosynthetic pathway. In physiological 96-h shake flask experiments, the optimal growth parameters resulted to be 28 °C and pH 7.0, at which a maximum biomass of 8.4 ± 0.5 g_cdw_/L, a melanin concentration of 0.74 ± 0.01 g/L (yield on biomass of 0.09 ± 0.01 g/g_cdw_ and productivity of 0.008 ± 0.001 g/L/h), and a final tyrosinase activity of 10.1 ± 0.1 U/mL were reached. The produced pigment was purified from the broth supernatant with a two-step purification process (75.0 ± 2.0% of purity with 65.0 ± 5.0% of recovery) and tested for its chemical, antioxidant, and photoprotective properties. Finally, characterization by UV-visible and FT-IR spectroscopy, elemental analyses, and mono- and bi-dimensional NMR suggested the eumelanin-like nature of the pigment.

## 1. Introduction

### 1.1. Melanins

Melanins are pigments widespread in the animal, plant, and microbial worlds whose colors change from reddish to brown or to dark brown [1,2,3,4]. They have peculiar chemical–physical characteristics like high thermal stability, good UV-visible light absorption capabilities, metal ion chelating ability, hybrid ionic-electronic conductance, antioxidant, and redox properties [5,6]. Thanks to these characteristics, they have been widely used in food, cosmetic, and textile industrial fields to manufacture UV-protective food packaging materials, sunscreen creams, lenses, colored cloths, paints, green (bio)electronic circuits, and filtering systems for water decontamination from heavy metals and toxins [3,6]. They also show good antimicrobial activity, biocompatibility, biostability, no cytotoxicity, and no antigenic response, so they are also nowadays widely employed in diverse biomedical fields as antioxidant and antitumor agents, as radio-protective molecules, in the preparation of 3D-bioprinted and biocompatible scaffolds for tissue regeneration, or in the manufacturing of nanoparticles then used as antiviral and antimicrobial drug carriers [7,8,9]. Nowadays, melanins are obtained by extraction and purification from animal tissues, as from the ink sac of cephalopods like cuttlefishes (*Sepia officinalis*) [10], or by chemical synthetic procedures. In the first case, the extraction process is very problematic and expensive due to the low quantities of pigment that could be recovered and the difficulties of the purification process because of possible interactions between the pigment and the proteins of the animal tissues. Synthetic melanin can be easily prepared by both enzymatic and chemical oxidative polymerization processes, but, in this case, the limiting step is the availability of the starting monomer precursors (i.e., 5,6-dihydroxyindole (DHI)). Both types of melanin are commercially available [11], but they are quite expensive. For all these reasons, in the last few years, the production of melanin by microorganisms has gained more and more attention as an alternative, sustainable procedure that is also easier to scale up [12].

### 1.2. Microbial Melanins

Bacteria and fungi can produce melanins starting from precursors like L-tyrosine or malonylCo-A [1,2]. L-tyrosine can be first converted by tyrosinase or laccase enzymes into L-3,4-dihydroxyphenylalanine (L-DOPA), then to 5,6-dihydroxyindole (DHI) and 5,6-dihydroxyindole-2-carboxylic acid (DHICA), the monomer precursors of eumelanins, or by p-hydroxyphenylpyruvate hydroxylase into homogentisic acid, the monomer precursor of pyomelanins. Pheomelanin, instead, is produced when L-tyrosine and/or L-DOPA are oxidized in the presence of L-cysteine, resulting in pigment with a red-yellow color. Five units of malonylCo-A can be used by a polyketide synthase to assembly the naphthalene backbone and then to form 1,8-dihydroxynaphthalene, the monomer precursor of fungal allomelanins, obtained by a laccase-promoted oxidative polymerization process [5,13,14,15]. Fungi belonging to *Auricularia*, *Aspergillus*, *Armillaria*, *Cryptococcus*, or *Pletorus* genus mainly produce melanin pigments as intracellular products in very long growth (up to 480–528 h) [16]. Among bacteria, strains of the *Proteus*, *Pseudomonas,* and *Streptomyces* genera are all able to produce melanins [14,16]. *Streptomyces* strains can produce eumelanin (or DOPA-melanin) and pheomelanin-like pigments (Figure S1). Although, so far, Streptomycetes have been very rarely employed as melanin producers in biotechnological processes, even if they are able to produce the pigment in a shorter time than fungi (120–168 h) and to release it in the growth medium as an extracellular product [17]. In the literature, only *Streptomyces cavourensis* RD8 [18], *Streptomyces cyaneofuscatus* [5], *Streptomyces glaucescens* NEAE-H [19], *Streptomyces kathirae* SC-1 [20], *Streptomyces lusitanus* DMZ-3 [2], *Streptomyces roseochromogenes* [21], *Streptomyces* sp. [12], and *Streptomyces* sp. ZL-24 [22] have been employed to produce melanins so far. The concentrations of the produced pigment greatly vary according to the strain, growth settings, and nutritional conditions [14]. Complex substrates of plant origin, like soy peptone, amylopectin, and even the rhizome fragments and the roots of a marine-origin grass plant, the egagropili of *Posidonia oceanica* [23], have been used as nutrients and supplemented in *Streptomyces* media to enhance melanin production. Also, complex animal-derived substrates, like casein or beef extract, or metal ions, and/or the L-tyrosine as precursor, have been provided in the growth media to better produce the pigment [2,16,19,20,21,22]. The production of melanin by *Streptomyces nashvillensis* DSM 40314, instead, has never been investigated so far, although this strain, isolated from the soil, is well noted to produce antibiotics like tetrodecamycin and bellenamine [24]. Besides, the influence of growth conditions, like pH and temperature, on the growth, on the melanin production, and on the activities of the enzymes involved in the pigment synthetic pathway, has never been investigated for this strain. In this paper, *Streptomyces nashvillensis* DSM 40314 was tested for the first time as a melanin-producing strain. The optimal conditions for both growth and melanin production were explored in physiological shake flask studies by exploring different temperatures (26, 28, and 30 °C) and pH values (6.0 and 7.0) on a medium containing glucose, malt extract, and yeast extract. The activity of the secreted tyrosinase enzyme was also investigated under different growth conditions and correlated with the melanin production. The obtained melanin, released in the clarified broth supernatant, was purified by using a two-step procedure, then chemically characterized and determined by UV-visible, Fourier-transform infrared (FT-IR), nuclear magnetic resonance NMR spectroscopy, and elemental analyses.

## 2. Materials and Methods

### 2.1. Materials

Medium components like yeast and malt extracts were purchased from Himedia Laboratories (Modautal, Germany), while glucose, (NH_4_)_2_SO_4_, NaH_2_PO_4_·H_2_O, Na_2_HPO_4_, NaCl, the synthetic melanin standard, and all the salts used for preparing the buffers, acid, base, and solvents used for the melanin precipitation, purification, and chemical characterization were provided by Sigma-Aldrich (Milano, Italy). L-DOPA for the tyrosinase assay was purchased from TCI (Tokio, Japan). Chemicals required for the melanin structural characterization by UV-visible absorbance and FT-IR analyses were purchased from Sigma-Aldrich (Milano, Italy).

### 2.2. Microorganism and Media

*Streptomyces nashvillensis* DSM 40314 was purchased from DSMZ (Braunschweig, Germany) and initially grown in a 1-L shake flask containing 200 mL of GYA medium (glucose (20.0 g/L), yeast extract (20.0 g/L), (NH_4_)_2_SO_4_ (2.0 g/L), NaH_2_PO_4_·H_2_O (5.8 g/L), Na_2_HPO_4_ (8.2 g/L) at pH 7.0), at 28 °C and under constant agitation of 250 rpm in a rotary air shaker (ISF-1-W, Kühner, Birsfelden (Basel), Switzerland) [25]. After 48 h of growth, the culture was centrifuged at 4 °C and 5000 rpm (Avanti J-20XPI, Beckman Coulter, Brea, CA, USA) and then resuspended in fresh GYA medium with 20% (*v*/*v*) glycerol to prepare cell stock solutions, according to a previously reported procedure [25]. The stocks were then stored at −80 °C. Bacterial growth was carried out in GEM III N medium at different pH conditions (6.0 or 7.0) (glucose (12.0 g/L), yeast extract (6.0 g/L), malt extract (30.0 g/L), with a buffer phosphate made of NaH_2_PO_4_·H_2_O (11.9 g/L), Na_2_HPO_4_ (1.9 g/L) for pH 6.0 or NaH_2_PO_4_·H_2_O (5.8 g/L), and Na_2_HPO_4_ (8.2 g/L) for pH 7.0) [25]. The media, devoid of glucose and Na_2_HPO_4,_ was sterilized by autoclaving at 120 °C for 20 min (ALFA-junior, PBI International, Milano, Italy). Solutions of glucose and Na_2_HPO_4_, sterilized by filtration with 0.22 µm membranes (Merck Millipore, Guyancourt, France), were added later to the autoclaved media.

### 2.3. Shake Flask Experiments

Physiological studies were performed at pH 6.0 or 7.0 and at different temperatures (26, 28, and 30 °C) in 250-mL shake flasks containing 50 mL of GEM III N medium, inoculated each with 100 µL of the glycerol stock solutions. Experiments were run in triplicate for 96 h under a constant agitation of 250 rpm on a rotary air shaker (ISF-1-W, Kühner, Birsfelden (Basel), Switzerland). Throughout the growth, broth samples of 4 mL were withdrawn at different time points to determine the dry cell weight of the biomass, the melanin production, and the secreted tyrosinase activity. Dry cell biomass was determined by filtering 2 mL of the broth samples on 0.22 μm polypropylene membranes (Merck Millipore, Guyancourt, France); the filtrates were collected, and the mycelium recovered on the membranes was then washed with a physiological saline solution and dried at room temperature up to the point when the dry cell biomass weight did not vary anymore [25]. Samples of culture broth were centrifuged at 4 °C at 5000 rpm for 20 min (Avanti J-20XPI, Beckman Coulter, Brea, CA, USA), and the supernatants were used to determine the melanin production by UV absorbance at 220 nm and the activity of the secreted tyrosinase enzyme. Duplicate cultures, inoculated with 400 µL of the glycerol stock solutions and grown for 96 h in 1-L shake flasks containing 200 mL of GEM III N medium at pH 7.0, at 28 °C, and under a constant agitation of 250 rpm in a rotary air shaker (ISF-1-W, Kühner, Birsfelden (Basel), Switzerland), were run to produce melanin that was then characterized for its chemical stricture and its properties. At the end of the cultivation time, culture broths were centrifuged at 4 °C and 5000 rpm for 20 min (Avanti J-20XPI, Beckman Coulter, Brea, CA, USA), and the supernatants were pooled to purify melanin.

### 2.4. Melanin Purification

The produced melanin was purified in a two-step process by acidic precipitation. A 5 M HCl solution was added to 400 mL of supernatant to reach pH 1.5, according to previously reported protocols [14,21]. The samples were kept at 4 °C overnight and then centrifuged at 4 °C and 5000 rpm for 20 min (Avanti J-20XPI, Beckman Coulter, Brea, CA, USA). The precipitated melanin was then washed three times with MilliQ water, recovered each time by centrifugation as described above, and then dried at room temperature. A portion of the precipitated dried melanin (about 15.0%) was then further washed with a 5 M HCl solution under stirring conditions and at room temperature for 5 h, using a slightly modified protocol [12,20]. This wash was performed to remove the eventual presence of proteins and nucleic acids bonded to the pigment. The sample was then centrifuged at 4 °C and 4500 rpm for 20 min (Avanti J-20XPI, Beckman Coulter, Brea, CA, USA), and the residual precipitated melanin was washed again three times with MilliQ water, dried as described above, and further used for characterization.

### 2.5. Tyrosinase Activity Assay

The extracellular tyrosinase activity was assayed on 1.0 mL of supernatant samples concentrated 5- or 10-fold by centrifugation at 12,000 rpm (Z216 MK, Hermle Labortechnik GmbH, Wehingen, Germany) using 3 kDa filter devices (Merck Millipore, Guyancourt, France for 12 min at 4 °C. L-DOPA was used as a substrate, according to a previously reported method [26]. The concentrated sample containing the enzyme (50 µL) was added to a freshly prepared solution (950 µL) of 2 mM L-DOPA in 13 mM KH_2_PO_4_ buffer at pH 6.5. The reaction of oxidation of L-DOPA to dopaquinone, performed by the secreted tyrosinase, was followed by measuring the kinetics of absorbance at 280 nm for 10 min at 25 °C (Spectrophotometer DU800, Beckman Coulter, Brea, CA, USA). One unit of tyrosinase was defined as the amount of enzyme that gives an increase of 0.001 unit of absorbance per minute [26].

### 2.6. Melanin Quantification

The concentration of melanin in shake flask supernatants was determined by spectrophotometric analysis by measuring the UV absorbance of the samples at 220 nm (Spectrophotometer V-530, Jasco, Tokio, Japan). A calibration curve was established in the range of 0.0005 to 0.01 g/L with the synthetic melanin standard after its dissolution in 0.1 M NaOH. To determine the melanin concentration in the supernatant samples, the initial measured absorbance of the growth medium was subtracted from the absorbance of each sample. The purity of the dried precipitated and purified melanin samples, obtained during the purification process, was calculated by determining the concentration of the melanin by measuring its absorbance at 220 nm after dissolving the samples in a defined volume of MilliQ water. Full UV-visible spectra, between 200 and 400 nm of the synthetic melanin standard of the 96 h supernatant cultures of the precipitated and purified melanin were also recorded during the purification process. In all cases, a 0.1 M NaOH solution was used as a blank.

### 2.7. Melanin Colorimetric Analysis

Colorimetric analyses were carried out on microfiltered (with 0.22 µm membranes; Millipore, France) broth supernatant samples at time 0 h and at the end (96 h) of the cultivations performed at pH 6.0 or pH 7.0 and at 26, 28, and 30 °C. Analyses were carried out using a Pump-Probe configuration by light absorption in diffuse reflection using a MAYA 2000 pro (Ocean Insight, Oxford, UK) spectrophotometer. The light source was a halogen lamp (CIE standard illuminant D65) with an emission spectrum in the range of 400 to 1000 nm. Before measurements, the device was calibrated using a white ceramic disk and a black trap portion. The commercial software OceanView 2.0 (Ocean Insight, Oxford, UK), with which the device is supplied, was used for the acquisition of the reflected spectra, the colorimetric data, and for basic operations. The measurements were performed by immersing the probe in the supernatant solutions. The colorimetric values of each sample were estimated by determination of their dominant wavelength (λ_DOM_) and of the x and y chromaticity coordinates that were then inserted in a CIE diagram 1931 using the free GoCIE V2 software (GoCIE (iitr.ac.in), developed by Dr. K. R. Justin Thomas, Department of Chemistry, Indian Institute of Technology Roorkee, India, 2009).

### 2.8. Melanin Structural Characterization

#### 2.8.1. Melanin Elemental Analysis

The elemental composition (C, H, and N % w) of the purified melanin (6.6 mg) was estimated using a PerkinElmer 2400 CHNSO elemental analyzer (PerkinElmer Instruments, Shelton, CT, USA).

#### 2.8.2. Melanin Analysis by Fourier-Transform Infrared (FT-IR) Spectroscopy

The purified melanin (1.3 mg) was mixed with KBr powder (199.0 mg) in an agate mortar, grounded, and then pressed in the form of a translucent disc to be scanned by Fourier-transform infrared spectroscopy (FT-IR) using a FT-IR-4700 instrument (Jasco, Tokio, Japan). Spectra were recorded in triplicate in the 4000–700 cm^−1^ range using 600 scans and a resolution of 2.0 cm^−1^. The synthetic melanin standard was analyzed under the same conditions.

#### 2.8.3. Melanin Analysis by Nuclear Magnetic Resonance (NMR) Spectroscopy

Both the precipitated (36.4 mg) and purified melanin samples (27.4 mg) were dissolved in DMSO-d6 as solvent and analyzed by NMR spectroscopy. ^1^H NMR spectra were recorded with a Bruker DRX-400 MHz instrument (Bruker Corporation, Billerica, MA, USA), whereas ^1^H,^1^H COSY experiments were run at 400.1 MHz using standard pulse programs.

### 2.9. Melanin Chemical Characterization

#### 2.9.1. Melanin Solubility and Metal Chelating Activity

The purified melanin was tested in terms of solubility and metal chelating ability, according to previously reported procedures [20,21]. The solubility was checked by preparing 10 g/L solutions of the pigment in MilliQ water or in 1 M NaOH, 1 M HCl, 1 M NaH_2_PO_4_, methanol, ethanol, acetone, and DMSO. The metal ion chelating ability of the purified melanin, instead, was tested by observing an eventual precipitate formation after preparing solutions of the pigment (1.0 g/L) containing 10 mM of Na^+^ (as NaCl), K^+^ (as KCl), Ca^++^ (as CaCl_2_), Mg^++^ (as MgCl_2_), Fe^+++^ (as FeCl_3_), Cu^++^ (as CuSO_4_·5H_2_O), Ni^++^ (as NiSO_4_·6H_2_O), or Zn^++^ (as ZnSO_4_·7H_2_O).

#### 2.9.2. Melanin Thermal Stability and Resistance to UV-Visible Light Exposure

Solutions of the purified melanin (0.1 g/L in 1 M NaOH) were prepared to test the pigment thermal stability and its resistance to UV or visible light radiations by heating the samples at 40, 60, 80, and 100 °C for 6 h or by exposing them under sun light or under the light of an UV lamp for 6 h (Hg source lamp, 250–350 nm, SterilGARD III advance, The Baker company, Sanford, ME, USA), according to previously described methods [20,21]. The absorbance values of the samples were measured at 220 nm (Spectrophotometer V-530, Jasco, Tokio, Japan) at the beginning of each test and then every two hours up to 6 h. The thermal stability and the UV or visible light radiation resistance were calculated using the following formula:

% = [1 − ((Abs_t0h_ − Abs_txh_)/(Abs_t0h_))] × 100 (1)

where Abs_t0h_ was the absorbance value of the melanin solutions at time 0 h and Abs_txh_ was the absorbance value after a time x (like after t 2 h, t 4 h, or 6 h) of heating at different temperatures or of UV-visible light exposure.

#### 2.9.3. Redox Behavior and Antioxidant Activity

The purified melanin was also tested for its properties of redox behavior by dissolving 0.1 g/L of the pigment in solutions of H_2_O_2_ or Na_2_S_2_O_3_ prepared at different percentages (0.1, 0.5, 1.0, 1.5, or 3.0%), and the samples were incubated for 48 h at room temperature under constant agitation of 100 rpm in a rotary air shaker (ISF-1-W, Kühner, Birsfelden (Basel), Switzerland), as previously reported [20]. The absorbance of the samples was measured at 220 nm (Spectrophotometer V-530, Jasco, Tokio, Japan) at time zero and after 48 h, and then the percentages of oxidability or reducibility were calculated according to the following formula:

% = [(Abs_t0h_ − Abs_t48h_)/(Abs_t0h_)] × 100 (2)

where Abs_t0h_ was the initial absorbance value of the melanin in the different H_2_O_2_ or Na_2_S_2_O_3_ solutions, whereas Abs_t48h_ was the value of the same solutions at 48 h. The antioxidant scavenging activity was determined after mixing 3 mL of a solution containing hydroxyl radicals (obtained by mixing in sequence 1 mL of 8.8 mM H_2_O_2_, 1 mL of 9.0 mM FeSO_4_, and 1 mL of 9.0 mM 4 hydroxybenzoic acid in 95% ethanol) with 1 mL of purified melanin solutions at concentrations between 0.2 and 1.0 g/L, as previously described [22]. A control solution was also prepared by mixing 1 mL of MilliQ water with 3 mL of the hydroxyl radical-containing solution. The mixtures were incubated for 30 min at 37 °C and 200 rpm, and then the hydroxylation of 4-hydroxybenzoic acid into 3,4-dihydroxybenzoate was measured at 510 nm (Spectrophotometer V-530, Jasco, Tokio, Japan). The percentage of antioxidant activity (%η) towards the hydroxyl radicals was calculated according to the following formula:

%η = [1 − (Abs_ms_ − Ab_sc_)/(Ab_sc_)] × 100 (3)

where Abs_ms_ was the absorbance value of the samples, while Ab_sc_ was the absorbance value of the control solution after incubation.

### 2.10. Data and Statistical Analysis

All the results reported in this manuscript are averaged values of three independent experiments, as calculated by using a Microsoft Office Excel 2007 program (Microsoft, Redmond, WA, USA), together with the standard deviation values. Comparisons between groups of data were also performed with the t-student test, and they were considered significantly different if the *p* values were lower than 0.05.

## 3. Results

### 3.1. Shake Flask Experiments

**Figure 1:**
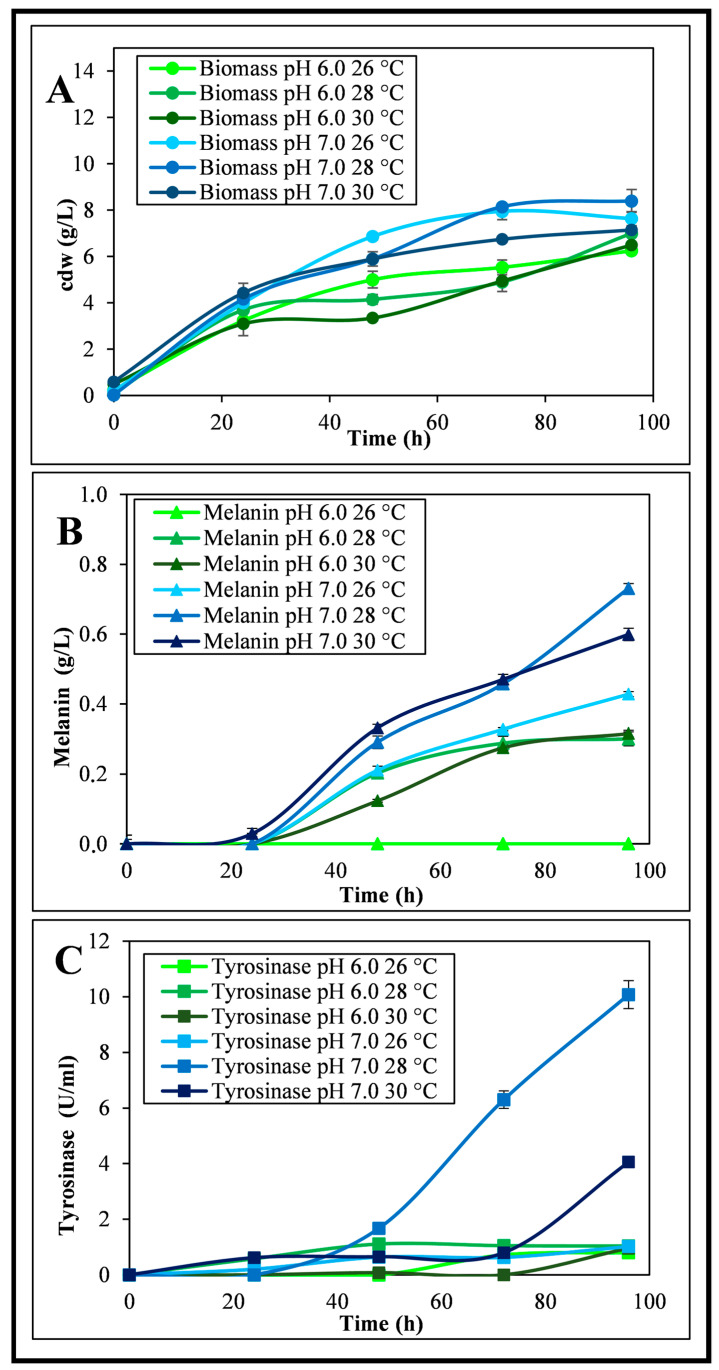
*S. nashvillensis* growth in shake flasks on GEM III N medium at different pH and temperature conditions up to 96 h: kinetics of biomass formation (**A**), melanin production (**B**), and secreted tyrosinase activity (**C**).

**Figure 2:**
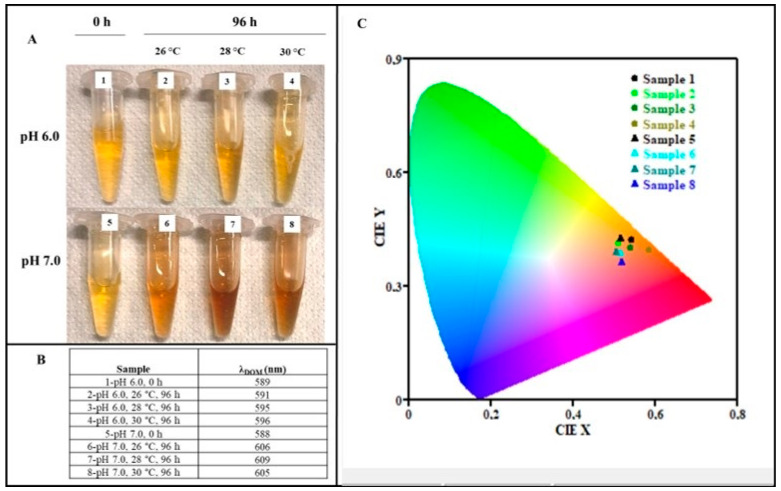
Colorimetric analyses of the broth supernatant samples of *S. nashvillensis* shake flask growth performed at different pH and temperature conditions: pictures of the samples (1 mL) at the beginning of the growth (0 h, at pH values 6.0 and 7.0—samples 1 and 5, respectively) and at the end of the growth (96 h, at pH 6.0 and 26, 28, and 30 °C—samples 2, 3, and 4, respectively; and at pH 7.0 and 26, 28, and 30 °C—samples 6, 7, and 8, respectively) (**A**) and their dominant wavelength values (λ_DOM_) in the visible spectrum (**B**). The representation of the x and y coordinates of all the samples in the CIE diagram 1931 shows a change in color from the light yellow-orange color of the initial supernatant samples (samples 1 and 5) towards a dark pink-orange color of the 96-h supernatant sample obtained at pH 7.0 and 28 °C (sample 7), at which the higher melanin production was determined (**C**).

Physiological shake flask studies were run to verify in which conditions the strain *Streptomyces nashvillensis* DSM 40314 would be able to produce extracellular melanin. To select the best pH and temperature conditions for both biomass formation and pigment production, the bacteria were grown on GEM III N medium at pH 6.0 or 7.0 and at temperature values of 26, 28, or 30 °C for 96 h (Figure 1). The kinetics of microbial growth and the final biomass values changed according to the conditions used (Figure 1A,B). At pH 6.0 and 26 °C, the biomass reached the final value of 6.3 ± 0.1 g_cdw_/L, but no melanin was produced (Figure 1A,B). By changing the pH to 7.0 at the same temperature, the final biomass reached the value of 7.6 ± 0.1 g_cdw_/L, and the strain was able to produce melanin up to a concentration of 0.43 ± 0.05 g/L, with a yield on biomass of 0.06 ± 0.04 g/g_cdw_ and a productivity of 0.004 ± 0.001 g/L/h. The activity of extracellular tyrosinase was determined in the supernatant at different time points of the cultures grown at pH 6.0 or 7.0 and 26 °C. For the growth at pH 6.0 and 26 °C, an activity due to the secreted tyrosinase was detected only starting from the 72th hour of growth, up to a final value of 0.8 ± 0.1 U/mL, while at pH 7.0 and 26 °C, an activity due to the secreted tyrosinase was determined since the 24th hour of growth, up to a final value of 1.0 ± 0.1 U/mL (Figure 1C). Other experiments were performed at 28 and 30 °C, and at these temperatures, the growth rates and the final biomass values were higher at pH 7.0 than at pH 6.0 and slightly higher at 28 °C than at 30 °C. Melanin production was also higher at pH 7.0 than at pH 6.0 and higher at 28 °C than at 30 °C. As a matter of fact, at pH 6.0 and 28 °C, the final biomass was 7.0 ± 0.6 g_cdw_/L, while the melanin production was 0.30 ± 0.02 g/L (yield on biomass of 0.04 ± 0.02 g/g_cdw_ and a productivity of 0.003 ± 0.001 g/L/h). This value was higher than the null production obtained at pH 6.0 and 26 °C, but 30% lower than the one obtained at pH 7.0 and 26 °C (Figure 1A,B). In these conditions, the secreted tyrosinase activity reached 1.0 ± 0.1 U/mL, a value similar to the one reported for the growth at pH 7.0 and 26 °C (Figure 1C). At the conditions of pH 7.0 and 28 °C, the final biomass reached 8.4 ± 0.5 g_cdw_/L, and the pigment concentration was 0.74 ± 0.01 g/L, with a yield on biomass of 0.09 ± 0.01 g/g_cdw_ and a productivity of 0.008 ± 0.001 g/L/h (Figure 1A,B). This melanin value was 2.3-fold higher than the one obtained at pH 7.0 and 26 °C. A higher kinetics of tyrosinase activity was also observed with a final value of 10.1 ± 0.1 U/mL, 10-fold higher than the activity reported for the end points of the growths at pH 7.0 and 26 °C or pH 6.0 and 28 °C (Figure 1C). In the growth performed at pH 6.0 and 30 °C, the final biomass was 6.5 ± 0.3 g_cdw_/L, while the melanin production was 0.31 ± 0.01 g/L (yield on biomass of 0.05 ± 0.03 g/g_cdw_ and a productivity of 0.003 ± 0.001 g/L/h), similar to the one obtained at pH 6.0 and 28 °C but with slower kinetics of production (Figure 1A,B). The tyrosinase activity reached a maximum of 1.0 ± 0.4 U/mL, a value similar to the one reported for the growth at pH 6.0 and 28 °C as well (Figure 1C). At the conditions of pH 7.0 and 30 °C, the final biomass and the pigment concentration were 7.2 ± 0.3 g_cdw_/L and 0.59 ± 0.01 g/L, respectively, with a yield on biomass of 0.08 ± 0.01 g/g_cdw_ and a productivity of 0.006 ± 0.001 g/L/h (Figure 1A,B), while the secreted tyrosinase activity reached a final value of 4.0 ± 0.1 U/mL (Figure 1C), lower than the one determined for the growth at pH 7.0 and 28 °C. Taking into consideration all these results, the conditions of pH 7.0 and 28 °C resulted in the best ones for both growth, secreted tyrosinase activity, and thus melanin biosynthesis. A change in the color of the starting medium was visibly observed in some supernatant samples due to the possible production of the pigment, with a darker shade noted for the 96 h supernatant of the growth at pH 7.0 and 28 °C (Figure 2A); thus, colorimetric analyses were also performed in the visible light range (Figure 2B,C). The colorimetric values of each sample were estimated by determining their dominant wavelength values (λ_DOM_) and the x and y chromaticity coordinates, which were then inserted in a CIE diagram 1931. The 96 h supernatant samples of the shake flask growth performed at pH 6.0 and at 26, 28, or 30 °C showed dominant wavelength values (591, 595, and 596 nm, respectively) slightly higher (up to 1.2%) than the values measured for the initial growth medium supernatant (589 nm) (Figure 2B). While the final dominant wavelength of the supernatant of the growth performed at pH 7.0 and 28 °C showed a wider change (up to 3.6%) than its initial broth supernatant sample (588 nm) up to 609 nm, a value slightly higher than the ones observed for the 96-h supernatants of the growth at pH 7.0 at 26 and 30 °C (606 and 605 nm, respectively) (Figure 2B). By inserting the obtained x and y chromaticity coordinates in the CIE diagram 1931, a clear change in color was determined from the light yellow-orange color of the initial supernatant samples (0 h) towards a dark pink-orange color of the 96 h supernatant samples of the runs performed at pH 7.0 and 28 °C (Figure 2C).

### 3.2. Melanin Purification and Structural Characterization

**Figure 3:**
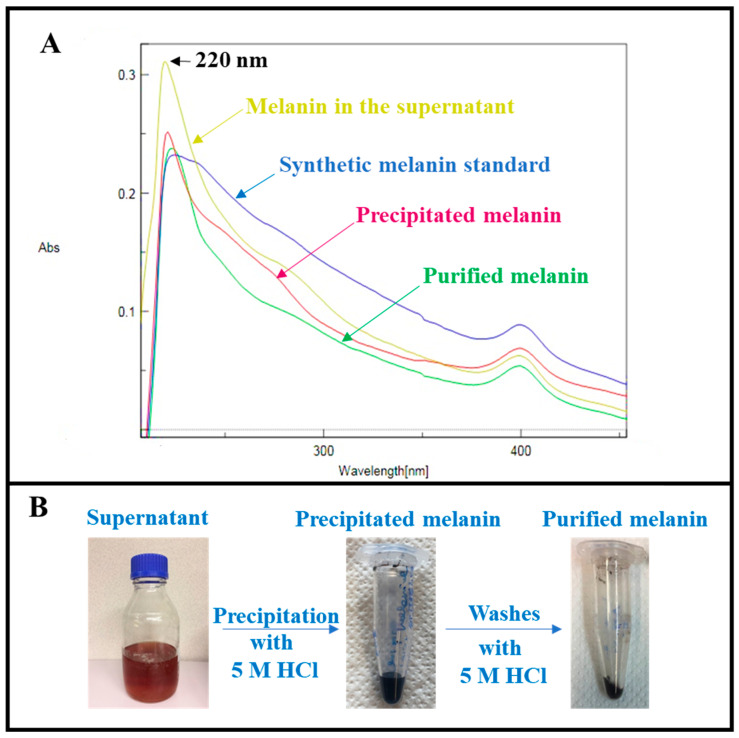
Superposition of UV-visible spectra of the synthetic melanin standard and of the melanin produced by *S. nashvillensis* as present in the broth supernatant (yellow line), after a first step of precipitation (red line) and after a further step of purification (green line) (**A**), obtained following a simple two-step purification procedure (**B**). The maximum peak at 220 nm is indicated by the arrow.

The melanin produced by *Streptomyces nashvillensis* on the GEM III N medium in the best growth conditions, at pH 7.0 and 28 °C, was purified and then structurally characterized by elemental analyses, FT-IR, and mono- and bi-dimensional NMR. UV-visible spectra were also recorded to further characterize the produced pigment and to follow its purification process. Spectra of the synthetic melanin standard and of the pigment present in the culture broth supernatant at the end of the growth were acquired and compared (Figure 3A). Both samples showed a similar absorption profile with the typical monotonic decay over the entire UV-visible spectrum, with a maximal absorbance at 220 nm. Here, a shoulder was visible at 280 nm, indicative of phenolic residues, and another peak was seen at 400 nm (Figure 3A). The melanin produced was first precipitated from the broth supernatant by the addition of a 5 M HCl solution; approximately 67.2 ± 2.8% of melanin was recovered (Figure 3A,B), a value similar to that previously reported for the melanin produced by *S. roseochromogenes* purified with the same procedure [21]. Despite this purification procedure, the precipitated melanin showed a very low purity (39.8 ± 0.7%) and a UV-visible spectrum with a high absorption in the range of 260–280 nm (Figure 3A), which indicated proteins and nucleic acid contaminations were still eventually bound to melanin by electron-affinity interactions [12,20]. To disrupt these bindings and thus to further purify the pigment, part of the precipitated melanin (about 15.0%) was then washed again with the 5 M HCl solution, under stirring conditions. After this treatment, the purified melanin showed a two-fold higher purity (75.0 ± 2.0%, with a final recovery of 65.0 ± 5.0%) and an UV-visible spectrum with a sharper monotonic decay in the range of 260–280 nm (Figure 3A,B). This purified melanin was then structurally characterized by elemental analysis, FT-IR, and mono- and bi-dimensional NMR spectroscopies. The elemental analysis of the purified melanin (Table 1) showed a high content of nitrogen (9.8%), compatible with a eumelanin-like pigment or a DOPA-melanin.

**Figure 4:**
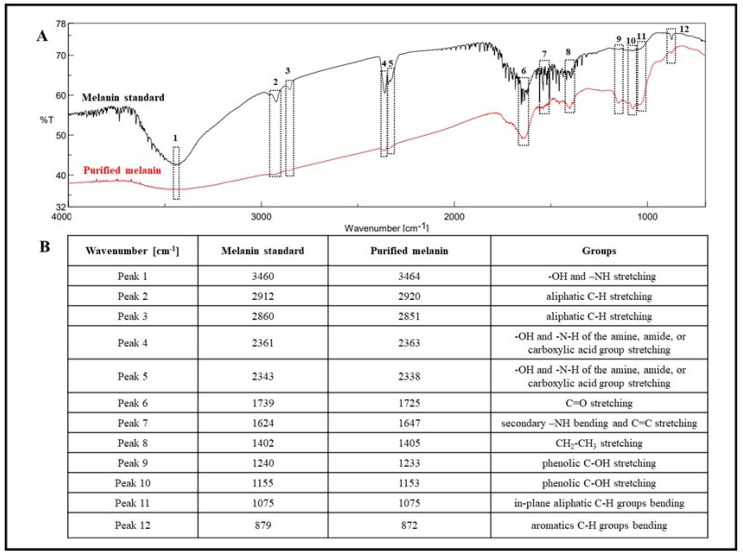
Overlaid FT-IR spectra of the synthetic melanin standard and of the purified melanin produced by *S. nashvillensis* (**A**) and the wavenumbers of the twelve different signals identified by the analysis (**B**).

**Figure 5:**
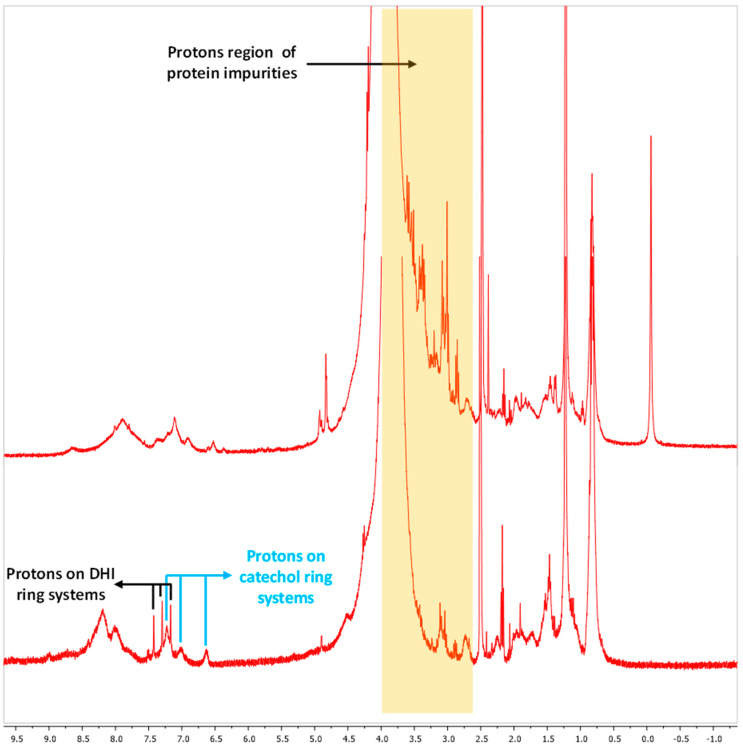
^1^H NMR spectra (DMSO-d6) of the precipitated (upper trace) and the purified melanin pigment (lower trace) produced by *S. nashvillensis*.

**Figure 6:**
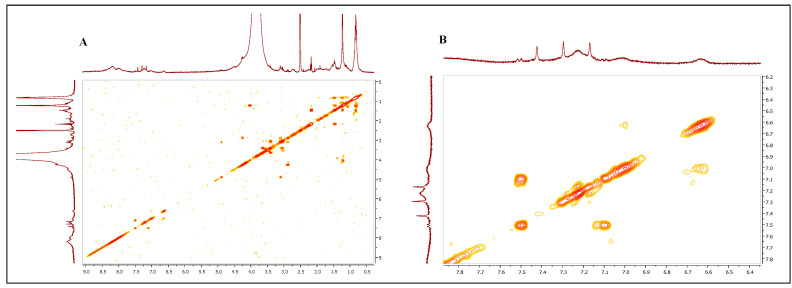
^1^H,^1^H COSY spectrum (DMSO-d6) of the purified melanin pigment (**A**). Expansion of the aromatic protons region (**B**).

To further characterize the pigment, FT-IR analyses of the purified melanin were performed, and 12 different signals were identified and compared with the signals of the synthetic melanin standard (Figure 4A,B). The first strong, broad peak, centered at 3464 cm^−1^ indicated the stretching and the presence of both -OH and -NH groups (peak 1), while the small, weak bands (peaks 2 and 3) at 2920 and 2851 cm^−1^ were due to the stretching vibration of aliphatic C-H groups. The broad signals around 2363 and 2338 cm^−1^ (peaks 4 and 5) could have been attributed to the stretching vibrations (O-H and N-H) of the amine, amide, or carboxylic acid groups present in the indolic groups (Figure 4A,B). The multiple signals around 1725 cm^−1^ (peaks 6) were attributed to the C=O stretching of quinone or carboxylic acid groups. The peaks observed at around 1647 cm^−1^ (peak 7) were considered due to the bending of secondary NH and to the stretching of the aromatic C=C groups (Figure 4A,B). The multiple signals around 1405 cm^−1^ (peak 8) were due to the CH_2_-CH_3_ bending, which is considered characteristic of the melanin pigment. The small peaks at 1233 and 1153 cm^−1^ (peaks 9 and 10) were considered due to the stretching of phenolic C-OH groups (Figure 4A,B). The last peaks at 1075 and 872 cm^−1^ (peaks 11 and 12) were due to the bending of in-plane aliphatic and aromatic CH groups, respectively, which are characteristic of the melanin pigment as well (Figure 4A,B). All these spectroscopic data of the purified pigment produced by *Streptomyces nashvillensis* correlated with those of the synthetic melanin standard and helped to identify the pigment again as having an eumelanin-like structure (Figure 4A,B). The NMR analyses carried out both on the precipitated and the purified melanin revealed that the two-step treatment with HCl removed almost completely the protein fraction associated with the pigment, as evident from the low number of protons signals in the 2.6–4 ppm region detected in the purified sample (Figure 5). Moreover, in the aromatic protons region, the signals of protons of the catechol ring system at 6.64, 7.01, and 7.22 ppm, coupled with each other as from the ^1^H,^1^H COSY spectrum (Figure 6), and of the 5,6-dihydroxyindole ring system at 7.17, 7.30, and 7.41 ppm were clearly discernible. These data not only denoted the efficiency of the purification protocol but also supported the structural assignment of the pigment, like a eumelanin-type mixed indolic/phenolic polymer.

### 3.3. Melanin Chemical Characterization

The purified melanin showed a dark brown color (Figure 3B), and it was immediately soluble in 1.0 M NaOH but slowly soluble in MilliQ water and DMSO, and completely insoluble in 1.0 M HCl, methanol, ethanol, acetone, and ethyl acetate (Table 2). Purified melanin was not able to chelate Na^+^, K^+^, Ca^++^, and Mg^++^ ions, but it was able to chelate Fe^+++^, Cu^++^, Ni^++^, and Zn^++^ metal ions. The pigment also showed very low oxidability and reducibility in the range from 1.56% to 8.45% and 0.45 to 5.79%, respectively, although values significantly changed (* *p* < 0.05), moving from the lower to the higher concentrations of H_2_O_2_ and Na_2_S_2_O_3_ solutions. In contrast, melanin solutions from 0.2 to 1.0 g/L were shown to exhibit a very high antioxidant activity from 93.5 to 96.9% towards hydroxyl radicals without any significant difference (* *p* < 0.05). Furthermore, the pigment was stable at temperatures between 40 and 100 °C, retaining 80.4 to 53.0% activity, although significant differences were found by increasing the temperature. The pigment was also highly resistant to UV and visible light exposure (83.4 and 80.4% of resistance, respectively, with no significant differences) (Table 2).

## 4. Discussion

### 4.1. Melanin Production by Streptomycetes

In their late growth or stationary phase, *Streptomyces* strains are well known to produce bioactive-specialized metabolites, including antibiotics and pigments, that have numerous interesting properties and, thus, potential industrial applications [17]. From a biotechnological point of view, the industrial production of pigments by *Streptomyces* species might be limited by the low concentrations, production yields, and productivities, as well as by the long and time-consuming processes to obtain them. However, in contrast with fungi that produce melanin as intracellular secondary metabolites, Streptomycetes produce these pigments extracellularly, facilitating their recovery in downstream purification processes [21]. It is noteworthy that a wise strain selection, a deep investigation of the best growth conditions, such as the optimal pH and temperature, and a modulation of the medium composition and the strain nutritional requirements are all key factors in developing a biotechnological process for secondary metabolite production by *Streptomyces* strains [14]. In the literature, temperatures between 26 °C and 30 °C and pH values of 6.0 or 7.0 were generally employed to produce melanin by *Streptomyces* strains, as in the case of *Streptomyces kathirae* SC-1 (pH 6.0 and 28 °C) [20], *Streptomyces* sp. (pH 7.0 and 30 °C) [12], and *Streptomyces roseochromogenes* (pH 6.0 and 26 °C) [21]. But in all these papers, the authors did not initially check the best growth conditions for each strain in physiological studies in order to optimize melanin production. Preliminary physiological studies were only performed in the cases of *Streptomyces cavourensis* RD8 [19] and *Streptomyces* sp. ZL-24 [22], and the best growth conditions for these species were established to be pH 7.0 and 30 °C and pH 6.0 and 30 °C, respectively. The activity of secreted tyrosinase was tested in the case of *Streptomyces* sp. ZL-24 [22] in different growth conditions, by changing the medium composition in terms of carbon and nitrogen sources and salts, as well as diverse pH and temperature values, in order to try to correlate this activity to the melanin production.

### 4.2. Melanin Production by Streptomyces nashvillensis DSM 40314

In this paper, for the first time, the possibility of employing *Streptomyces nashvillensis* DSM 40314 as a producer of extracellular melanin was investigated. Physiological studies in shake flasks were performed to optimize growth, melanin production, and assess the tyrosinase activity at different pH and temperature conditions selected in ranges classically used in diverse *Streptomyces* biotechnological processes [21,25]. The kinetics of bacterial growth were influenced more by the pH than by the temperature, with the final biomass always higher at pH 7.0 than pH 6.0 in all temperature conditions. However, the range of variation of the final biomasses was rather limited among the six conditions since the values ranged from about 6.3 to 8.4 g_cdw_/L. The best final biomass for *S. nashvillensis* (approximately 8.0 g_cdw_/L) was obtained at pH 7.0 and 28 °C and was similar to that obtained for *S. roseochromogenes* when grown on the same medium (GEM III N) in shake flasks at pH 6.0 and 28 °C [21]. Both pH and temperature conditions turned out to be critical for the activity of the secreted tyrosinase and for melanin production as well. The tyrosinase activity varied from a minimum value of 0.8 ± 0.1 U/mL at pH 6.0 and 26 °C to a maximum of 10.1 ± 0.1 U/mL at pH 7.0 and 28 °C. This maximal value of tyrosinase activity was in the same range as the reported intensity for *S. roseochromogenes* tyrosinase when grown on the same GEM III N medium [21]. In general, the higher the tyrosinase activity, the better the melanin production, although discrepancies were noted between the quantity of melanin produced and the intensities of activity detected in the different growth conditions. These differences might depend on the fact that we tested only the secreted enzyme and not the intracellular tyrosinase [27]. The synthesis, the activation from apotyrosinase, and the subsequent secretion in the external medium of tyrosinase in *Streptomyces* strains are regulated by two genes, *melC1* and *melC2*, present in an operon [27,28,29,30,31,32,33,34,35,36,37]. The effective secretion of the tyrosinase in the medium is mediated by the MelC1 protein, although mutants defective in the *melC1* gene and in the secretion of the tyrosinase have been found. In these cases, the tyrosinase remains in the cells, where it is still active in producing melanin [27,28,29,30,31,32,33,34,35,36,37]. From our data, it seems that some pH and temperature values mainly influenced the secretion of the enzyme that is not detectable in the supernatant samples, but it worked intracellularly to produce melanin as well. This could explain why a relevant amount of melanin was found in our experiments, even if low intensities of tyrosinase activities have been detected in the same conditions. The increase in tyrosinase activity in the late stationary phase, as detected in some cases, could be either due to a cell lysis phenomenon or a slower release of the enzyme in the medium. The best conditions for growth (pH 7.0 and 28 °C) and secreted tyrosinase activity also resulted in the best ones for melanin biosynthesis. The maximal melanin production by *S. nashvillensis* was approximately 0.74 g/L, a production 1.4 fold higher than that reported for *Streptomyces roseochromogenes* (approximately 0.53 g/L), grown on the same medium in shake flasks at pH 6.0 and 26 °C [21], and 2.8 fold higher than that reported for *Streptomyces lusitanus* DMZ-3 (about 0.26 g/L) [2], and 3.9 folds and 8.9 folds higher than that reported for *Streptomyces* sp. ZL-24 (about 0.19 g/L) [22] and for *Streptomyces cavourensis* RD8 (0.08 g/L) [19], respectively. In the best growth conditions, melanin production and its release in the growth medium were associated with a visible color change in the starting medium from the light yellow-orange color towards a dark pink-orange color. Melanin is frequently analyzed by elemental UV-visible, FT-IR, and NMR spectroscopic analyses, but, as far as we know, this is the first time when the release of a melanin pigment produced by a microbial strain was detected by colorimetric analyses. The extracellularly released pigment produced by *S. nashvillensis* was also easily recovered and purified, thanks to a simple two-step procedure that resulted in a purity of 75% of the compound. Furthermore, UV-visible spectrum analyses revealed a sharp form of the typical monotonic decay curve, similar to those already reported for the melanin pigment produced by other strains like *Streptomyces lusitanus* DMZ-3 [2], *Streptomyces glaucescens* NEAE-H [19], and *Streptomyces* sp. ZL-24 [22]. NMR data also confirmed the efficiency of our purification to remove the proteins associated with the pigment. Our two-step protocol of purification, which involved the use of an acid solution to precipitate the clarified broth supernatant and then washing the melanin, was simpler and more straightforward than the other previously reported protocols, which involved multiple steps of precipitation by acid solution, resuspension in NaOH solution, treatment with proteases, and several washes [20]. Our protocol also led to the obtainment of melanin with a high grade of purity similar to that obtained from reverse-phase chromatographic-based purification [21]. Our process also kept intact the structure of the pigment, as confirmed by the structural NMR analyses. Both NMR and FT-IR analyses detected several signals typical of the indolic/phenolic nature of the produced melanin. In particular, the FT-IR signals reported in this paper were almost identical to those already reported for the pigment produced by other *Streptomyces* strains, such as *Streptomyces lusitanus* DMZ-3 [2], *Streptomyces glaucescens* NEAE-H [19], and *Streptomyces* sp. ZL-24 [22]. The elemental analysis suggested a composition typical of eumelanin, similar to that reported for the animal-origin eumelanin-like standard (%C = 52.5%, %H = 6.0%, %N = 13.3%, %O = 27.5%) [18] and the pigment produced by *Streptomyces cavourensis* RD8 [18] and *Streptomyces cavourensis* SV 21 [38], but different from those reported for the pigments produced by *Streptomyces* sp. [12]. In the latter, the presence of sulfur (around 2.12%), instead, clearly indicated a pheomelanin-like structure. The pigment produced by *S. nashvillensis* showed also has many interesting, characteristic chemical properties that have already been reported for the melanins produced by other *Streptomyces* strains [12,20], such as high thermal stability, UV light resistance, antioxidant activity, and heavy metal-chelating activity. Its eumelanin-like structure could open many different perspectives for its industrial applications.

## 5. Conclusions

In conclusion, for the first time, it was reported in this paper that the strain *Streptomyces nashvillensis* DSM 40314 can produce extracellular melanin up to 0.74 g/L in the optimal growth conditions of 28 °C and pH 7.0 on a glucose, yeast extract, and malt extract-based medium. The pigment produced is eumelanin-like melanin, slowly soluble in water, and able to resist harsh temperature conditions. Its chemical characteristics make it suitable for many industrial applications as a pigment, metal chelator, UV protector, and antioxidant agent. All these characteristics could open innovative possibilities for using this pigment in biodegradable multifunctional films and coatings for food packaging and food safety applications, as well as, eventually, in environmental decontamination processes.

## Figures and Tables

**Table 1:** Elemental analysis of the purified melanin by *S. nashvillensis*.

Melanin by *S. nashvillensis*	
Carbon (C, % w)	60.1
Hydrogen (H, % w)	8.3
Nitrogen (N, % w)	9.8
Others (O, % w)	21.8

**Table 2:** Solubility, chelating ability, oxidability, reducibility, antioxidant activity, thermal stability, and UV and visible light exposure resistance of the purified melanin produced by *S. nashvillensis*.

Solubility	Solvent	Yes/No
	H_2_O	Partly
	1.0 M NaOH	Yes
	1.0 M HCl	No
	Methanol	No
	Ethanol	No
	Acetone	No
	DMSO	Partly
Chelating ability	Metal ion	Yes/no
	10.0 mM Na^+^	No
	10.0 mM K^+^	No
	10.0 mM Ca^++^	No
	10.0 mM Mg^++^	No
	10.0 mM Fe^++^	Yes
	10.0 mM Cu^++^	Yes
	10.0 mM Ni^++^	Yes
	10.0 mM Zn^++^	Yes
H_2_O_2_ (0.1–3.0%) oxidability	h	%
	48 h	From 1.56 ± 0.05 to 8.45 ± 0.03
Na_2_S_2_O_3_ (0.1–3.0%) reducibility	h	%
	48 h	From 0.41 ± 0.01 to 5.79 ± 0.01
Antioxidant activity	Melanin (g/L)	%η
	0.2	93.5 ± 0.1
	0.4	95.2 ± 0.1
	0.8	95.1 ± 0.1
	1.0	96.9 ± 0.1
Thermal stability	°C	%
	40 °C	80.4 ± 0.1
	60 °C	77.8 ± 0.1
	80 °C	60.2 ± 0.1
	100 °C	53.0 ± 0.1
	h	%
UV light resistance	6 h	83.4 ± 0.1
VIS light resistance	6 h	80.4 ± 0.2

## Data Availability

The data presented in this study are available on request from the corresponding author.

## Supplementary Materials

The following supporting information can be downloaded at: https://www.mdpi.com/article/10.3390/microorganisms12020297/s1, Figure S1: Structures of different types of melanin produced by *Streptomyces* strains.

## References

[1] Carletti G., Nervo G., Cattivelli L. (2014). Flavonoids and melanins: A common strategy across two kingdoms. Int. J. Biol. Sci..

[2] Madhusudhan D., Zainab Mazhari B.B., Dastager S.G., Agsar D. (2014). Production and cytotoxicity of extracellular insoluble and droplets of soluble melanin by *Streptomyces lusitans* DMZ-3. BioMed Res. Int..

[3] Solano F. (2017). Melanin and melanin-related polymers as materials with biomedical and biotechnological applications-cuttlefish ink and mussel foot proteins as inspired biomolecules. Int. J. Mol. Sci..

[4] Glagoleva A.Y., Shoeva O.Y., Khlestkina E.K. (2020). Melanin pigment in plants: Current knowledge and future perspectives. Front. Plant Sci..

[5] d’Ischia M., Napolitano A., Pezzella A., Meredith P., Buehler M. (2020). Melanin biopolymers: Tailoring chemical complexity for materials design. Angew. Chem. Int. Ed..

[6] Al Khatib M., Harir M., Costa J., Baratto M.C., Schiavo I., Trabalzini L., Pollini S., Rossolini G.M., Basosi R., Pogni R. (2018). Spectroscopic characterization of natural melanin from *Streptomyces cyaneofuscatus* strain and comparison with melanin enzymatically synthesized by tyrosinase and laccase. Molecules.

[7] Mostert A.B. (2021). Melanin, the what, the why and the how. Polymers.

[8] Pezzella A., Barra M., Musto A., Navarra A., Alfè M., Manini P., Parisi S., Cassinese A., Criscuolo V., d’Ischia M. (2015). Stem cell-compatible eumelanin biointerface fabricated by chemically controlled solid state polymerization. Mater. Horiz..

[9] Manini P., Lucci V., Lino V., Sartini S., Rossella F., Falco G., Chiappe C., d’Ischia M. (2020). Synthetic mycomelanin thin films as emergent bio-inspired interfaces controlling the fate of embryonic stem cells. J. Mater. Chem. B.

[10] Mbonyiryivuze A., Omollo I., Ngom B.D., Dhlamini S.M., Park E., Maaza M. (2015). Natural dye sensitizer for grätzel cells: Sepia melanin. Phys. Mater. Chem..

[11] Sigma Aldrich. https://www.sigmaaldrich.com.

[12] Li C., Ji C., Tang B. (2018). Purification, characterization, and biological activity of melanin from *Streptomyces* sp.. FEMS Microbiol. Lett..

[13] Funa N., Funabashi M., Ohnishi Y., Horinouchi S. (2005). Biosynthesis of hexahydroxyperylenequinone melanin via oxidative aryl coupling by cytochrome P-450 in *Streptomyces griseus*. J. Bacteriol..

[14] Pralea I.-E., Moldovan R.-C., Petrache A.-M., Ilies M., Heghes S.-C., Ielciu I., Nicoară R., Moldovan M., Ene M., Radu M. (2019). From extraction to advanced analytical methods: The challenges of melanin analysis. Int. J. Mol. Sci..

[15] Ahn S.-Y., Jang S., Sudheer P.D.V.N., Choi K.-Y. (2021). Microbial production of melanin pigments from caffeic acid and L-tyrosine using *Streptomyces glaucescens* and FCS-ECH-expressing *Escherichia coli*. Int. J. Mol. Sci..

[16] Tran-Ly A.N., Reyes C., Schwarze F.W.M.R., Ribera J. (2020). Microbial production of melanin and its various applications. World J. Microb. Biotechnol..

[17] Barbuto Ferraiuolo S., Cammarota M., Schiraldi C., Restaino O.F. (2021). Streptomycetes as platform for biotechnological production processes of drugs. Appl. Microbiol. Biotechnol..

[18] Dholakiya R.N., Kumar M.A., Mody K.H. (2017). Production and characterization of melanin from *Streptomyces cavourensis* strain RD8 using response surface optimization. Environ. Pollut. Prot..

[19] El-Ahmady El-Naggar N., El-Ewasy S.M. (2017). Bioproduction, characterization, anticancer and antioxidant activities of extracellular melanin pigment produced by newly isolated microbial cell factories *Streptomyces glaucenscens* NEAE-H. Sci. Rep..

[20] Guo J., Rao Z., Yang T., Yang T., Man Z., Xu M., Zhang X. (2014). High-level production of melanin by a novel isolate of *Streptomyces kathirae*. FEMS Microbiol. Lett..

[21] Restaino O.F., Scognamiglio M., Mirpoor S.F., Cammarota M., Ventriglia R., Giosafatto C.V.L., Fiorentino A., Porta R., Schiraldi C. (2022). Enhanced *Streptomyces roseochromogenes* melanin production by using the marine renewable source *Posidonia oceanica* egagropili. Appl. Microbiol. Biotechnol..

[22] Wang L., Li Y., Li Y. (2019). Metal ions driven production, characterization and bioactivity of extracellular melanin from *Streptomyces* sp. ZL-24. Int. J. Biol. Macromol..

[23] Restaino O.F., Giosafatto C.V.L., Mirpoor S.F., Cammarota M., Hejazi S., Mariniello L., Schiraldi C., Porta R. (2023). Sustainable exploitation of *Posidonia oceanica* sea balls (Egagropili): A Review. Int. J. Mol. Sci..

[24] Ikeda Y., Naganawa H., Kondo S., Takeuchi T. (1992). Biosynthesis of bellenamine by *Streptomyces nashvillensis* using stable isotope labeled compounds. J. Antibiot..

[25] Restaino O.F., Marseglia M., De Castro C., Diana P., Forni P., Parrilli M., De Rosa M., Schiraldi C. (2014). Biotechnological transformation of hydrocortisone to 16α-hydroxy hydrocortisone by *Streptomyces roseochromogenes*. Appl. Microbiol. Biotechnol..

[26] Masterman D., Redding K. (2010). Advanced Biology with Vernier: Experiments for AP and College General Biology.

[27] Claus H., Decker H. (2006). Bacterial tyrosinases. Syst. Appl. Microbiol..

[28] Leu W.M., Chen L.Y., Liaw L.L., Lee Y.H. (1992). Secretion of the *Streptomyces* tyrosinase is mediated through its trans-activator protein, MelC1. J. Biol. Chem..

[29] Chen L.Y., Chen M.Y., Leu W.M., Tsai T.Y., Lee Y.H. (1993). Mutational study of *Streptomyces* tyrosinase trans-activator MelC1. MelC1 is likely a chaperone for apotyrosinase. J. Biol. Chem..

[30] Lee Y.H., Chen B.F., Wu S.Y., Leu W.M., Lin J.J., Chen C.W., Lo S.C. (1988). A trans-acting gene is required for the phenotypic expression of a tyrosinase gene in *Streptomyces*. Gene.

[31] Liaw L.L., Lee Y.H. (1995). Histidine residues 102 and 117 of MelC1 play different roles in the chaperone function for *Streptomyces* apotyrosinase. Biochem. Biophys. Res. Commun..

[32] Chen L.Y., Leu W.M., Wang K.T., Lee Y.H. (1992). Copper transfer and activation of the *Streptomyces* apotyrosinase are mediated through a complex formation between apotyrosinase and its trans-activator MelC1. J. Biol. Chem..

[33] Tseng H.C., Lin C.K., Hsu B.J., Leu W.M., Lee Y.H., Chiou S.J., Hu N.T., Chen C.W. (1990). The melanin operon of *Streptomyces antibioticus*: Expression and use as a marker in gram-negative bacteria. Gene.

[34] Endo K., Kamo K., Hosono K., Beppu T., Ueda K. (2001). Characterization of mutants defective in melanogenesis and a gene for tyrosinase of *Streptomyces griseus*. J. Antibiot..

[35] Pandey B.P., Lee N., Kim B.G. (2017). Effect of Extracellular Tyrosinase on the Expression Level of P450, Fpr, and Fdx and Ortho-hydroxylation of Daidzein in *Streptomyces avermitilis*. Appl. Biochem. Biotechnol..

[36] Lee N., Kim E.J., Kim B.G. (2012). Regioselective Hydroxylation of trans-Resveratrol via Inhibition of Tyrosinase from *Streptomyces avermitilis* MA4680. ACS Chem. Biol..

[37] Tsai T.Y., Lee Y.H. (1998). Roles of copper ligands in the activation and secretion of *Streptomyces* tyrosinase. J. Biol. Chem..

[38] Wibowo J.T., Kellermann M.Y., Petersen L.-E., Alfiansah Y.R., Lattyak C., Schupp P.J. (2022). Characterization of an insoluble and soluble form of melanin produced by *Streptomyces cavourensis* SV 21, a sea cucumber associated bacterium. Mar. Drugs.

