# Supplementary figures and images for: Biotechnological Production and Characterization of Extracellular Melanin by Streptomyces nashvillensis
Source: Microorganisms. 2024 Jan 30;12(2):297. doi: 10.3390/microorganisms12020297 (PMC10892051; doi:10.3390/microorganisms12020297)

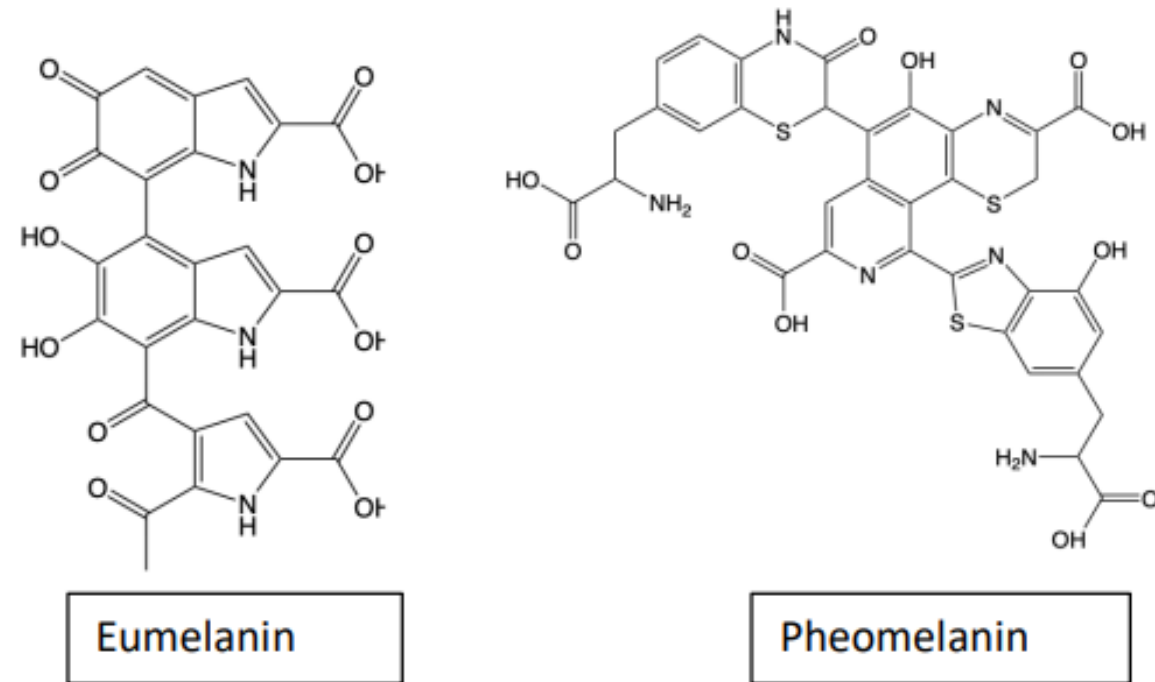

**Figure S1.** Structures of different types of melanin produced by *Streptomyces* strains.

Supplement: Supplementary file 1 [file microorganisms-12-00297-s001.zip › microorganisms-2812206-supplementary.pdf]
